# Anti-HCV reactive volunteer blood donors distribution character and genotypes switch in Xi'an, China

**DOI:** 10.1186/1743-422X-7-186

**Published:** 2010-08-10

**Authors:** Qiao-hong Yue, Xian-qing Zhang, Yu Shang, Yao-zhen Chen, Wen-li Sun, Min-quan Su, Shi-jie Mu, Xiao-ke Hao, Xing-bin Hu

**Affiliations:** 1 Department of Clinic Molecular Research Center& Clinic Diagnostic Laboratory, Xijing Hospital, Fourth Military Medical University, 17th Changlexi Road, Xi'an 710032, China; 2 Department of Blood Transfusion, Xijing Hospital, the Fourth Military Medical University,17th Changlexi Road, Xi'an 710032, China; 3 School of Electronic Information Engineering, Xi'an Technological University, xi'an 710032, China

## Abstract

HCV is prevailed in the world as well as in China. Blood transfusion is one of the most common transmission pathways of this pathogen. Although data of HCV infection character were reported during the past years, anti-HCV reactive profile of China donors was not fully clear yet. Furthermore, infection progress was found related to the HCV genotype. Different genotype led to different efficacy when interferon was introduced into HCV therapy. Here we provided character data of HCV infection in China blood donors from the year of 2000 to 2009. The infection rate in local donors was lower than general population and descended from 0.80% to 0.40% or so in recent years. About 83% HCV strains were categorized into genotypes 1b and 2a. But 1b subtype cases climbed and 2a subtype cases decreased. The current study threw more light on HCV infection of blood donors in China, at least in the Northern region.

## Background

Hepatitis C virus (HCV) infection rate is about 3% and more than 170 million people are currently infected by HCV in the world [1]. More than 3.5 million new sufferers annually occurred [2]. The situation is more serious in China because more than 50 million HCV cases located in this country [3]. This infection, mainly transmitted by blood transfusion in China, could progress to cirrhosis liver and hepatocarcinoma [4].

HCV is an enveloped virus with a single strand positive and non- fragment RNA. The genome of HCV is about 9 400 nucleotides, which encodes approximately 3 000 amino acids [5]. The high heterogenic nucleotides of HCV were confirmed and at least six different genotypes have generally been divided [6,7]. Furthermore, HCV quasispecies were clarified also according to more detailed HCV genome variation [8-10].

The distribution of HCV genotypes and subtypes depends on geographical location and race deference [2,7,8,11]. Type 1a, the first identified sequence, was popular in the United States, while Asia cases were observed also. About 10-30% HCV infection belonged to type 2a and 2b virus in the world. Type 2a and 2b pathogens prevailed in the North America, Europe, China and Japan. Type 2c strains occurred only in the North region of Italy. Type 3 viruses were more observed in India, Southeast Asia and Indonesia. But type 3a strain prevailed in drug users in the North Europe and United States and mixed with type 1a virus infection. Type 4 virus was mainly reported in the Mediterranean Sea country. More than 39.2% cases belonged to type 5 family in the South Africa. Type 6 virus infections concentrated in the Southwest of China, including Hong Kong and Taiwan region.

It is significant to discern HCV genotype because infection progress was found related to the nuclei acid sequence variation [12,13]. Different genotype led to different efficacy when interferon was introduced into HCV therapy [14]. Actually, HCV genotypes were regarded as an independent prediction factor in interferon administration [15]. Thus HCV genotyping research is necessary in clinic, including transfusion medicine.

Although HCV genotyping was once performed in China, the infection character and genotype distribution is not fully clear yet in blood donors [16-18]. Since gene sequence variation may lea possible failure in donor's screen test, it is imperative to obtain epidemiological data to decrease the blood transmission risk.

The aim of this study was to analyze the HCV epidemiology in local donor population in the past 10 years. The distribution and formulation of HCV infection was also clarified here. HCV genotypes of local blood donors were probed in the current study.

## Results

### Infection rate in the last 10 years decreased

**Figure 1:**
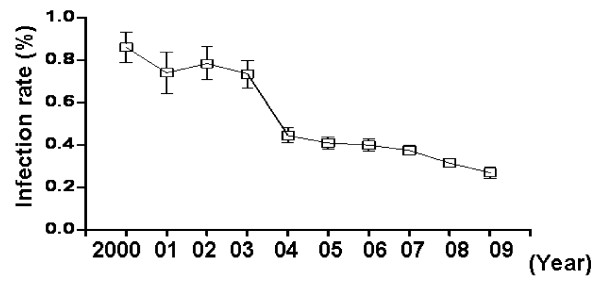
**HCV infection rate in blood donors from the year of 2000 to 2009**. Donors' peripheral blood serum were isolated and employed to ELISA to test reactive anti-HCV antibody. Bars represent the 95% confidence interval.

To determinate the HCV infection rate in local blood donors, ELISA was performed according to the standard donor peripheral blood test procedure. From the January of 2000 to the December of 2009, about 0.45% (1151/273203) blood donors were found with anti-HCV positive reaction. As shown in Fig. 1, the infection rate is higher in the early of the study period. In the year of 2001, 0.81% donors were anti-HCV reactive. From the year of 2004, anti-HCV reactive rate in donors decreased to 0.42% and kept stable in the followed years. In the year of 2010, HCV infection rate is 0.54% hitherto (117/21578, up to 31^st ^May, 2010).Those data implied that anti-HCV reactive donors decreased in the latest 5 years in local region.

### Most of the anti-HCV reactive donors were low-grade infection

**Figure 2:**
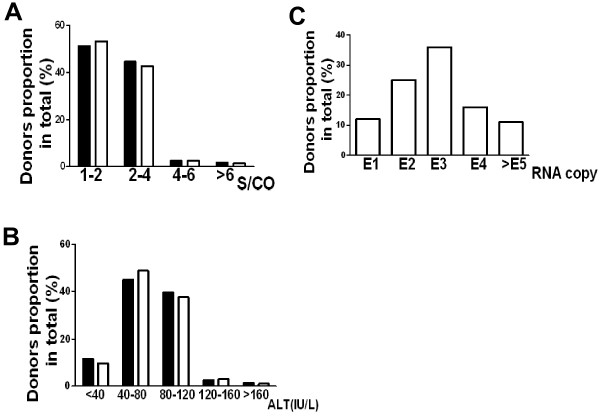
**HCV reactive profile of blood donors in represented (blank) and total samples (black)**. Represented and total samples from donors were administrated to standard ELISA, ALT and real-time PCR determination methods. The map was drawn according to different range respectively. A, S/CO value distribution of donors in anti-HCV antibody test; B, ALT value distribution of donors; C, viral load distribution of represented donors. E1:1 × 10^1^; E2:1 × 10^2^; E3:1 × 10^3^; E4:1 × 10^4^; E5:1 × 10^5^.

The relative value of sample absorbance and cutoff (S/CO) can somewhat reflect the degree of HCV infection. Thus we divided the 1151 anti-HCV reactive donors into different groups (Fig. 2A, black). More than 50% anti-HCV reactive donors' S/CO values were fewer than 2.0, while about 45% anti-HCV reactive donors' S/CO values were between 2.1 and 4.0. In ALT level measurement, more than 80% anti-HCV reactive donors' value was between 40 IU/L and 120 IU/L (Fig. 2B, black), while only a few donors' value was lower than 40 IU/L or higher than 120 IU/L. The 200 recruited donors for genotyping displayed similar distribution character (Fig. 2A and 2B, blank), which meant that the chosen sample pool, at least in part, could represent the total HCV infected donors in the current study. When HCV viral load detection was performed in the 200 recruited donors, about 61% samples' viral load were between 1 × 10^2 ^and 1 × 10^3 ^copies/ml, while 16.3% samples' viral load fell into 1 × 10^4 ^copies/ml (Fig. 2C). Altogether, these results suggested that most of the local anti-HCV reactive donors, without syndromes, had a low-grade infection when their blood collected.

### Genotypes distribution and changes in local region

**Figure 3:**
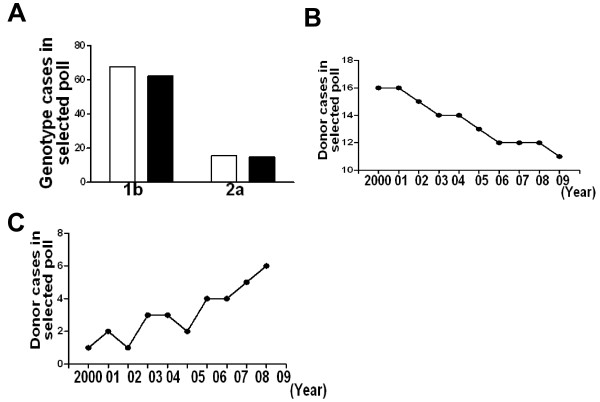
**Genotype distribution and switch in donors and patients**. Blood sample form 200 represented donors and 100 patients were genotyped. Then genotype 1b and 2a cases were counted. After that, rate of 1b and 2a cases were distributed in different years. A, genotype 1b and 2a comparison in donors (blank) and patients (black); B, genotype 1b distribution from the year of 2000 to 2009; C genotype 2a distribution from the year of 2000 to 2009.

To explore the HCV genotypes of local donors in the past 10 years, we chose 20 samples each year to perform genotyping. As shown in Table 1, 135 (67.5%) donors were 1b infection and 31 donors (15.5%) were 2a infection. The donor's age or sex was not associated with HCV infection. To be noted, 2 donors were mixed infection (1b and 2a) and 3 donors' infection genotype was not clarified. Furthermore, we compared 1b and 2a subtypes infection between donors and clinic confirmed patients. Results showed that 1b and 2a subtypes infection was no difference in these two groups (Fig. 3A). These data indicated that most of the local anti-HCV reactive donors infected with 1b and 2a subtypes virus.

**Table 1:** Genotype distribution in anti-HCV reactive donors from 200 represented samples

Genotype	Cases	Percentage (%)	Sex Ratio	Mean Age
1a	10	5.0	2.1	41 ± 10.1
1b	135	67.5	1.1	39 ± 11.6
2a	31	15.5	1.3	38 ± 8.9
2b	6	3.0	0.4	45 ± 12.3
3a	5	2.5	0.2	33 ± 11.4
3b	5	2.5	1.1	43 ± 7.1
6a	3	1.5	0.3	45 ± 14.3
1b+2a	2	1.0	0.8	34 ± 11.3
NC	3	1.5	0.7	29 ± 8.7

**Total**	200	100	0.9*	36 ± 9.7**

Sex Ratio was counted on the Number of males to that of females.

NC: Not clarified genotype.

*: Total sex ratio.

**:Total mean age.

Since 1b and 2a HCV virus were the prevailed strains in local infected donors, we are wondering whether the two main genotypes changed in the past years. As shown in Fig. 3, the cases of 1b genotypes decreased in local HCV infected donors, from 16 cases in the year of 2000 to 11 cases in the year of 2009 (Fig. 3B), while the cases of 2a genotypes increased in the last 10 years(Fig. 3C). Other genotypes did not change (data not shown). These results showed that in the past 10 years, the two main genotypes (1b and 2a) in local HCV infected donors underwent a switch, although not so markedly.

### ALT and viral load in follow up research

To monitor HCV infection progress after the preliminary analysis, we tracked some reactive donors, who donated blood from the year of 2000 to the year of 2007 and were genotyped also as mentioned above, in the following 2 years. These samples were negative also for other hepatitis virus. Consecutive ALT testing showed that the liver function of these donors infected with HCV, at least partly, impaired one year later or so after donation (Table 2). Especially, 1b genotype virus infected donors had a higher ALT level than that of other genotype virus infected donors in the follow up time (Table 2, *P *< 0.05). Virus load was serially quantitated by real-time PCR in the tracked donors. As shown in Table 3, the number of virus copies increased in all the donors. Once again, 1b genotype virus RNA was higher in the follow up period (*P *< 0.05). Altogether, these data suggested that 1b genotype infection led to more serious liver function impairment for local donors.

**Table 2:** ALT levels in the consecutive test of represented anti-HCV reactive donors

Donor Number	Genotype	Age	Sex	ALT (U.L^-1^)				
				6	12	18	24	(month)
NO.17	3a	48	F	30	41	48	57	
NO.26	1b	27	F	48	66	80	118	*
NO.28	1b	41	M	34	59	110	157	*
NO.43	2a	30	F	45	56	ND	105	
NO.59	1a	41	M	48	59	77	119	
NO.70	2b	50	M	55	62	90	115	
NO.74	2a	35	M	38	ND	71	93	
NO.77	1b	38	M	54	79	93	147	*
NO.93	NC	24	F	55	57	66	ND	
NO.96	1b	39	F	44	49	73	127	*

ND: not determined.*:*P *< 0.05 when compared to other genotypes.

**Table 3:** HCV load in the following test of represented antibody reactive donors

				Viral load (copies/ml)				
Donor Number	Genotype	Age	Sex	6	12	18	24	(month)
NO.17	3a	48	F	3.1E1	2.9E2	3.5E2	1.2E3	
NO.26	1b	27	F	4.2E2	3.9E2	2.5E3	1.6E4	*
NO.28	1b	41	M	4.5E1	4.9E2	6.5E3	2.5E4	*
NO.43	2a	30	F	3.7E2	5.9E2	ND	1.5E4	
NO.59	1a	41	M	4.4E2	3.8E2	3.5E3	1.9E4	
NO.70	2b	50	M	5.2E2	5.9E2	8.0E3	NC	
NO.74	2a	35	M	3.2E1	ND	4.0E3	7.4E3	
NO.77	1b	38	M	3.2E2	4.9E3	9.0E3	2.4E4	*
NO.93	NC	24	F	3.1E1	2.9E2	3.5E2	ND	
NO.96	1b	39	F	2.2E2	3.9E2	6.5E3	1.8E4	*

ND: not determined. *:*P *< 0.05 when compared to other genotypes.

## Discussion

Since blood transfusion was generally accepted as one of the main dissemination pathways, more and more rigour test procedure was employed to detect HCV in donors [18]. In the current study, HCV average infection rate (0.45%) in local donors was much lower than that of Chinese open population. One of the reasons might be the strict questionnaire before donation and a great number of carriers were ruled out. On the other hand, the questionnaire could not deny the possibility of low-grade infection, thus the value of S/CO and virus RNA in most anti-HCV reactive donors in the present study were not so high.

The infection rate decreased from the year of 2004, in which gold-fast strip test was used before blood collection in our blood bank. Actually, without gold-fast strip test, the infection rate was much close to Sosa-Jurado *et al *reports (0.81% *v.s*. 0.84%). Interestingly, they observed descending HCV infection rate also from the year of 2003 to 2006 and contributed this decrease to stringent questionnaire [11].

Genotyping of HCV is necessary for clinic treatment and care counseling [19,20]. It is also useful to monitor the virus strains distribution profile and identify risk factors involved with transmission [11,21]. Blood transfusion was one of the main dissemination pathways. Thus it is great significant to analyze HCV genotype distribution for the sake of infection control. Although bulks of data described HCV genotype distribution in donors from different region [7,8,11,16], the situation in different region of China is somewhat unknown. Thus according to our knowledge, the present study firstly displayed the HCV genotype distribution in local region.

Althougth NS5b region sequences is thought as the most reliable technique for subtyping, hitherto 5' non coding region of HCV was the one of the most conserved sequence and thus extensively adapted in sub-type discrimination, especially in clinic HCV investigation. Other HCV gene fragments can also be used for genotyping. But they are not so popular because of more possible mutation, uncertain PCR products, less convenience and much higher costs in clinic. Before better and mature genotyping strategy innovation, 5' non coding region of HCV is the feasible choice for clinic investigation.

As occurred in other Eastern Asia region, 1b and 2a subtype prevailed in local region although other subtypes were observed also [16,17,22]. What's more, we found that the main prevailed subtypes in patients and donors were no difference. Interestingly, 1b and 2a subtype in local HCV infected donors underwent a switch. Donors infected with 1b virus descended in the past years, while donors infected with 1b virus climbed. It is not clear yet why this switch happened. One of the possible machinery is the population mobility. In fact 1b virus infection was prevailed in the south region of China and 2a virus infection was prevailed in the north region of China [23]. Since local city is at the cross of south and north, the observed subtype switch was no strange.

To track the donor's HCV infection, we made a two-years follow up in some anti-HCV reactive donors. Bulks of evidence showed that 1b genotype could bring more serious damage to liver [24]. Since we have ruled out other hepatitis virus infection, our data was consistent with that postulation according to the ALT level in the followed time. Again, viral load measurement also confirmed that 1b HCV virus duplicated more quickly than other genotypes did, which contributed to the liver function damage [25].

## Conclusion

The current study provided data of HCV infection in China blood donors during the past ten years. The infection rate in local donors was lower than general population and descended in recent years. Genotypes were clarified in the represented donor sample pool and 1b subtype was the prevailed strains. But 1b and 2a genotype switched in the past years in local region. These results threw more light on HCV infection of blood donors in China, at least in the Northern region.

## Materials and methods

### Blood donor, sample collection and follow up

Volunteer blood donors from both urban and rural areas in Xi'an City, from January of 2000 to December of 2009, were recruited into current study. They were medically assessed and denied any known risk factors for viral infection listed in the questionnaire. 200 donors with HCV (20 donors each year), ruled out HBV infection, were asked to give peripheral blood samples for genotyping. The serum were isolated from the samples, then subpackaged and stored at -80°C before analysis. Ten samples from the chosen 200 donors (donated before the December of 2007 and negative for other hepatitis virus) were followed up in the next 2 years. The sampling, isolation and storage procedures were just like the mentioned above. The present study was approved by the Ethics Committee of Fourth Military Medical University and the informed consents were signed.

### ELISA for anti-HCV detection

Test of the anti-HCV antibody were performed by ELISA using automatic enzyme detection system (Tecan GroupLtd., Mannedorf, Switzerland) and commercial kit (InTec Products, Xiamen, China). Briefly, 96-well plates were coated with antigen. Donors' peripheral blood serum was isolated and added into wells by automatic enzyme detection system before incubation. The plates were subsequently washed 5 times with PBST, and then the horseradish peroxidase labeled mono-antibody was added. After incubation, followed the manufacturer's instructions, washed plates and developed colorant to determine the results with absorbance reader (Thermo Scientific, Wohlen, Switzerland).

### Alanine-aminotransferase (ALT) measurement

To measure the ALT level, donors' peripheral blood serum was isolated and employed to automatic biochemistry analyzer (Hitachi, Tokyo, Japan) with commercial Kit (Fousun Long March Medical Ltd., Shanghai, China).

### Nucleic acid assay of HCV viral load

RNA from donor's blood sample was prepared according to the manufacturers' manual (Qiagen, Hilden, Germany). In brief, 500 μl isolated serum was mixed with 500 μl TRIzol regent and extracted with chloroform and alcohol. After quantitation, reverse transcribed into cDNA using random primers was performed (Qiagen, Hilden Germany). After that, real time PCR was used to detect HCV RNA according the manufacture's manual (Daan Gene, Shenzhen, China) with standard controls [3,26]. Briefly, extracted RNA was measured with fluorescence labeled and self-quench probe and Perkin Elme PCR analyzer (PTC-200, Perkin Elmer, Covina, USA). The viral load used the copies/ml as the units.

### Genotyping

**Figure 4:**
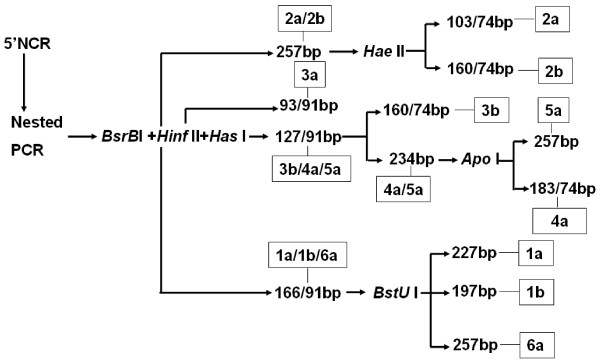
**The scheme of genotyping of HCV from recruited volunteer donors**. Samples were prepared and nested-PCR was performed. After that, serial restriction enzyme digestion was administrated to PCR products according to reports[7,8,20].

Genotyping was accomplished according to reported methods [7,8,27]. Briefly, Extracted RNA from the peripheral blood of 200 donors or 100 confirmed patients. Then the reverse transcription with random hexaprimers was carried out. After that, the seminested PCR of the 5' non-coding region with generic primers were performed. Amplicons were digested by restriction enzymes (Takara, Osaka, Japan). The digestion model, possible products and genotype categorization were schemed in Fig. 4.

### Statistical analysis

Differences between groups were statistically analyzed using SPSS 10.0.When *P *< 0.05, the difference was considered significant.

## Competing interests

The authors declare that they have no competing interests.

## Authors' contributions

YQH carried out the donor screen and drafted the manuscript. ZXQ participated in the real time PCR. SY performed statistics analysis. CYZ performed ALT analysis. SWL and SMQ carried out follow-up. MSJ, HXKand HXB predicated in the design of the study. All authors read and approved the final manuscript.
